# High Adiposity Is Associated With Higher Nocturnal and Diurnal Glycaemia, but Not With Glycemic Variability in Older Individuals Without Diabetes

**DOI:** 10.3389/fendo.2018.00238

**Published:** 2018-05-14

**Authors:** Raymond Noordam, Neline C. Huurman, Carolien A. Wijsman, Abimbola A. Akintola, Steffy W. M. Jansen, Stephanie Stassen, Marian Beekman, Ondine van de Rest, P. Eline Slagboom, Simon P. Mooijaart, Diana van Heemst

**Affiliations:** ^1^Section of Gerontology and Geriatrics, Department of Internal Medicine, Leiden University Medical Center, Leiden, Netherlands; ^2^Section Molecular Epidemiology, Department of Medical Statistics and Bioinformatics, Leiden University Medical Center, Leiden, Netherlands; ^3^Division of Human Nutrition, Wageningen University and Research, Wageningen, Netherlands; ^4^Institute for Evidence-Based Medicine in Old Age, IEMO, Leiden, Netherlands

**Keywords:** continuous glucose monitoring, body composition, adiposity, glycemia, glycemic variability

## Abstract

**Background:**

It is well known that adiposity is a risk factor for insulin resistance and type 2 diabetes mellitus. In the present study, we aimed to investigate the associations of measures of adiposity with indices of glycemia and of glycemic variability over a 72-h period in non-diabetic older adults.

**Methods:**

This cross-sectional study was conducted in non-diabetic individuals from the Active and Healthy Aging Study (*N* = 228), Switchbox (*N* = 116), and the Growing Old Together Study (*N* = 94). Body mass index (BMI) and waist circumference were measured, and indices of glycemia and glycemic variability were derived from continuous glucose monitoring (CGM) using the Mini-Med^®^ CGM system. Associations between adiposity and CGM were studied separately for the three cohorts, and derived estimates were subsequently meta-analyzed.

**Results:**

After meta-analyzing the results from the separate cohorts, individuals with a higher BMI had higher levels of glycemia. Individuals with BMI between 30 and 35 kg/m^2^ had 0.28 mmol/L [95% confidence interval (CI): 0.12–0.44] higher 72 h-mean glucose concentration, 0.26 mmol/L (0.10–0.42) higher diurnal glucose (6:00 a.m. to 0:00 a.m.), and 0.39 mmol/L (0.19; 0.59) higher nocturnal glucose (3:00 a.m. to 6:00 a.m.) than participants with a normal weight (BMI 18.5–25 kg/m^2^). However, no associations were observed between higher BMI and glycemic variability. Results for glycemia and glycemic variability were similarly observed for a high waist circumference.

**Conclusion:**

High adiposity associates with constant higher mean glucose levels over the day in non-diabetic older adults.

## Introduction

The prevalence of obesity, defined as a body mass index (BMI) above 30 kg/m^2^ (1, 2), is rapidly increasing worldwide, reaching pandemic proportions (3). Obesity is a generally known, and causal, risk factor for the development of insulin resistance and type 2 diabetes mellitus (T2DM) (4), and weight loss is generally associated with a reduced incidence of T2DM in individuals with impaired glucose tolerance (2). Nevertheless, in addition to overall adiposity, which is frequently measured with BMI, abdominal adiposity, which is frequently measured with waist circumference, has been shown to be a stronger predictor (and independent of BMI) for insulin resistance and future T2DM than BMI (5–7). Higher overall adiposity levels are associated with increased IL-6, TNF-α, and leptin expression (8, 9), and lower adiponectin expression (10, 11), which all could promote an insulin resistance state.

There is little insight into the dynamic aspects of glucose homeostasis related to obesity and increased adiposity, which might provide us with more (biological) insights as well as targets for interventions for disease prevention. Among individuals without T2DM, indices of both glycemia and glycemic variability, measures that both reflect the dynamic aspects of glucose homeostasis, were higher in older individuals compared with younger individuals (12). With respect to obesity, one cross-sectional study comprising 169 newly diagnosed T2DM patients of Chinese ancestry found evidence that a higher BMI was associated with a lower glycemic variability (13). However, studies on the association between adiposity and glycemia and glycemic variability in individuals without T2DM have not yet been performed. Information on glycemia and glycemic variability over a 24-h period can be obtained with continuous glucose monitoring (CGM), which measures blood glucose concentrations in the interstitial fluid while the participants can pursue normal daily activities (14). Although CGM has been previously shown to provide accurate results in normo-glycemic individuals for the purpose of research (15), to date, most research studies on CGM have focused on populations comprising participants with T2DM.

To provide additional insights into the association between adiposity and indices of glycemia and glycemic variability, we aimed to investigate the association between measures of body composition and indices of 24-h glycemia and glycemic variability in three independent study populations comprising older adults without T2DM.

## Materials and Methods

### Study Settings

The present, cross-sectional, study was embedded in the Active and Healthy Aging Study (“Actief en Gezond Oud,” AGO), Switchbox and Growing Old Together (GOTO) studies.

The AGO study aimed to investigate the effect of a web-based lifestyle intervention program with the intention to increase physical activity, on metabolic health. For this study, individuals aged 60–70 years living in the city of Leiden, the Netherlands, were recruited. Individuals with a history of T2DM, an active lifestyle, or a contraindication to increase physical activity were not included. In total, 243 individuals were enrolled and were randomized to either the intervention program or the control arm of the AGO study. The AGO study was registered in the Dutch Trial Register (http://www.trialregister.nl) as NTR3045.

The Switchbox Study aimed to investigate the neuro-endocrine mechanisms underlying maintenance of homeostasis in familial longevity. Individuals were enrolled from the ongoing, and larger, Leiden Longevity Study (16). Participants were eligible when their age was between 55 and 77 years and they had a stable BMI between 19 and 33 kg/m^2^. Participants were not eligible for participation in the Switchbox study if they had a fasting glucose above 7.0 mmol/L, if they had renal, hepatic, or endocrine disease, or if they used any medication known to influence lipolysis, thyroid function, glucose metabolism, GH/IGF-1 secretion, or any other hormonal axis. Furthermore, we did not include individuals who smoked, used more than 20 units of alcohol per day, or who had extreme diet therapies. In addition, participants who had a recent trans-meridian flight were not included as well. In the end, the total Switchbox Study population comprised 135 individuals.

The GOTO study aimed to investigate the effect of a combined physical activity and diet intervention on metabolic and metabolomic phenotypes. Similar to the Switchbox Study, participants were enrolled from the Leiden Longevity Study (16). Individuals of ages between 46 and 75 years and with a BMI between 23 and 35 kg/m^2^ were eligible to participate. Exclusion criteria were: treatment for T2DM, a fasting glucose level above 7.0 mmol/L, a weight change of more than 3 kg during the last 6 months, engagement in heavy/intensive physical activity (top sport or physically heavy work), any disease or condition that seriously affects body weight (e.g., cancer, heart failure, COPD), recent immobilization for >1 week, psychiatric or behavioral problems, use of thyroid mediation or immunosuppressive drugs, concurrent participation in any other intervention study or weight management program, or not having a general practitioner. In total, 163 individuals were enrolled in the GOTO study. For the present study, participants who already participated in Switchbox were excluded. The GOTO study was registered in the Dutch Trial Register (http://www.trialregister.nl) as NTR3499.

The designs and recruitment strategies of the three studies have been described in more detail elsewhere (12, 17–19). The three studies were carried out in accordance with the recommendations of the Helsinki Declaration. The protocol was approved by the medical ethical committee of the Leiden University Medical Center (LUMC), Leiden, the Netherlands. All three studies were performed within the LUMC. Written informed consent was obtained from all study participants.

### Study Design

The present study was conducted using a cross-sectional study design in participants without T2DM. One participant of the AGO study was excluded because of newly diagnosed diabetes mellitus (mean 24-h glucose ≥11.1 mmol/L) (20).

### Anthropometrics

Weight (in kilograms), height (in centimeters), and waist circumference (in centimeters) were measured at the study center of the LUMC by research nurses. BMI was calculated by dividing the weight (in kilograms) by height (in meters) squared. We used cut-off points according to the guidelines from the World Health Organization (1, 2) to group the participants. BMI was classified as “normal weight” (18.5–25 kg/m^2^), “overweight” (25–30 kg/m^2^), “obesity class I” (30–35 kg/m^2^), or “obesity class II” (>35 kg/m^2^). For waist circumference, we used gender specific cut-off points. A waist circumference ≤80 cm in women and ≤94 cm in men was classified as low waist circumference, and a waist circumference >88 cm in women and >102 cm in men was classified as high waist circumference. Participants between the cut-offs were classified as the middle group (1, 2).

### Glucose Measurements

For all participants, CGM was performed with the Mini-Med^®^ CGM system (Medtronic Minimed Inc., Northridge, CA, USA). For five consecutive days, interstitial glucose levels were monitored every 5 min with a glucose sensor (Sof-Sensor^®^, Medtronic, Minimed Inc., Northridge, CA, USA) inserted into the subcutaneous abdominal fat tissue. For calibration of the sensor, participants measured their capillary blood glucose four times a day by means of a finger prick. While continuing their normal daily activities, participants were asked to register their food intake, medication, and physical exercise during the study in a diary. In line with the instructions from the manufacturer, the first and fifth day of the measurements were excluded to maximize the accuracy of the data, leaving 3 days (covering 72 h) of data for the present study. Missing values of the CGM indices was mostly due to malfunction of the device, and thus random.

On the basis of the retrieved glucose trajectories, we calculated multiple indices of glycemia and glycemic variability for each participant. We calculated three indices of glycemia, notably 72-h mean glucose concentration, the mean diurnal glucose concentration (6:00 a.m. to 0:00 a.m.), and the mean nocturnal glucose level (3:00 a.m. to 6:00 a.m.), as being previously used (21–23). Indices of glycemic variability were the 72-h SD mean amplitude of glucose excursion (MAGE), and the mean of daily difference (MODD). The MAGE, which determines intraday glycemic variability, was calculated by a standardized algorithm (24). The MODD, which determines between-day glycemic variability, was calculated as the mean of the absolute difference of glucose values obtained at exactly the same time of the day from two consecutive days (25). These calculations for glycemia and glycemic variability have been validated in non-diabetic individuals before (15) and have already been used in previous studies (12, 14).

### Statistical Analyses

Characteristics of the study population are presented as the mean (SD) or as number (percentage), for the three cohorts in this study (AGO, Switchbox, and GOTO) separately as well as pooled.

We used multivariable linear regression analyses to study the associations between the obesity/waist circumference groups and the indices of glycemia and glycemic variability using STATA v12.0 (StataCorp LP, College Station, TX, USA). Participants in the lowest group of BMI (<25 kg/m^2^) and waist circumference (≤80 cm in women, ≤94 cm in men) were used as the reference population in the analyses on BMI and waist circumference, respectively.

All linear regression analyses were adjusted for age and sex. Analyses in Switchbox and GOTO were additionally corrected for familial relationships using robust SE. Additionally, in sensitivity analyses, we adjusted the associations between the two adiposity measures and the indices for glycemic variability for the 72-h mean glucose concentration. Sensitivity analyses were performed stratified for men and women. Results of the study populations were combined using a fixed effect inverse-variance weighted meta-analysis as implemented in the rmeta() statistical package for the R statistical environment (26). All results are presented as mean difference with respect to the reference group and with a 95% confidence interval (CI).

## Results

### Characteristics of the Study Population

For the present study, we used complete data from 438 participants of whom 228 participants were from AGO (226 in the analysis on waist circumference), 116 participants from Switchbox, and 94 participants from GOTO (Table 1). Pooled mean age was 64.7 (SD 4.6) years, and the study comprised for 55.3% of men. Pooled mean BMI was 27.7 (SD 4.0) kg/m^2^, and pooled mean waist circumference was 98.4 (SD 11.6) cm. Characteristics of the individual cohorts separately are presented in Table S1 in Supplementary Material.

**Table 1:** Characteristics of the total study population.

	*N* = 438
**Demographics**	
Age (years), mean (SD)	64.7 (4.6)
Men, *n* (%)	242 (55.3)
**Body composition**	
Body mass index (kg/m^2^), mean (SD)	27.7 (4.0)
Waist circumference (cm), mean (SD)^a^	98.4 (11.6)
**Measurements derived with continuous glucose monitoring**	
72-h mean glucose (mmol/L), mean (SD)	5.4 (0.6)
Nocturnal glucose (mmol/L), mean (SD)	4.7 (0.7)
Diurnal glucose (mmol/L), mean (SD)	5.6 (0.6)
MAGE, mean (SD)	2.3 (0.8)
72-h SD, mean (SD)	1.0 (0.3)
MODD, mean (SD)	0.9 (0.3)

*MAGE, mean amplitude of glycemic excursions; MODD, mean of daily differences*.

*^a^Missing for two participants*.

### Adiposity and Indices of Glycemia

**Figure 1:**
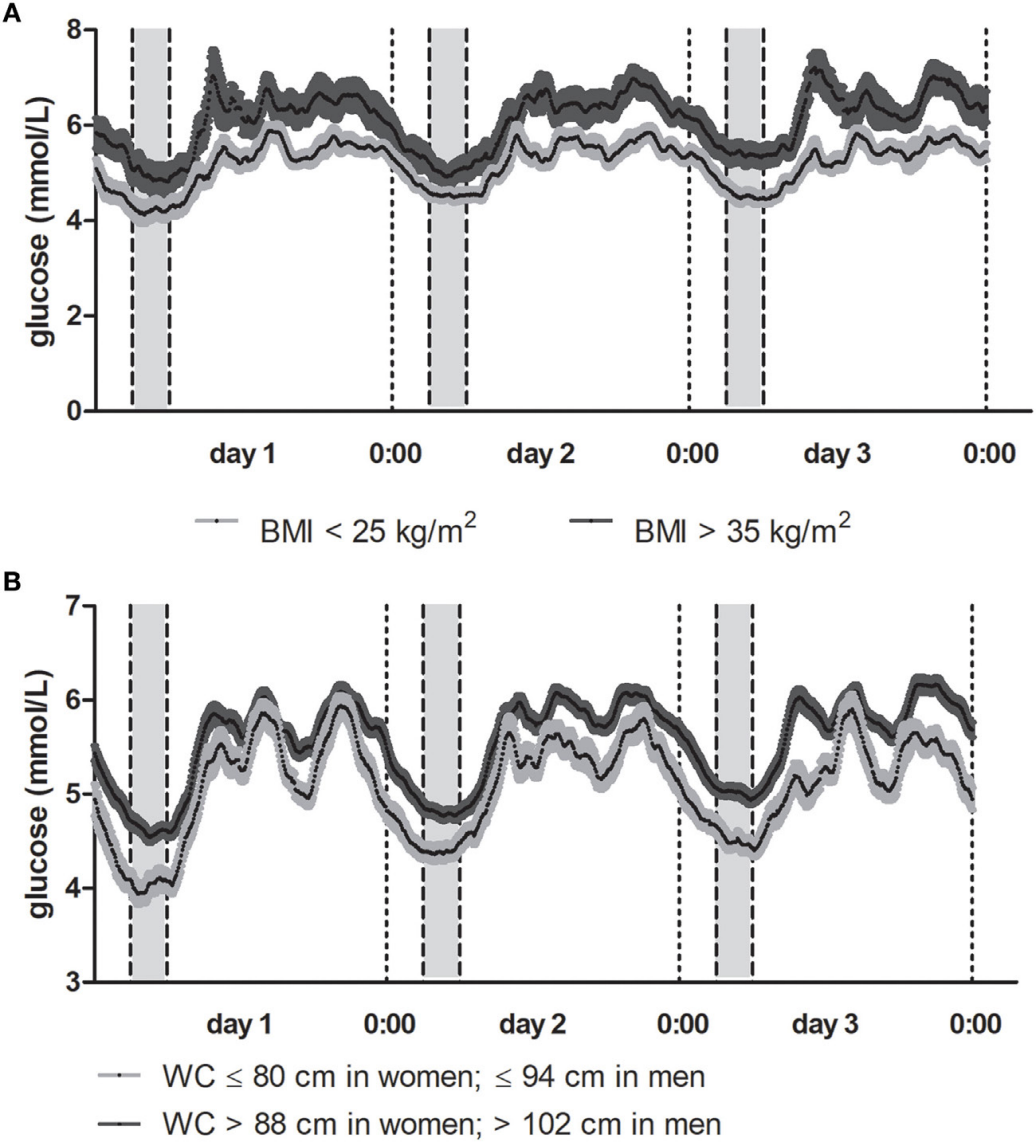
**(A)** Mean glucose trajectory in participants by body mass index (BMI). Results depicted as the mean (SE) glucose concentrations per 5-min interval for a 72-h period. In dark gray, mean (SE) glucose trajectory for participants with BMI > 35 kg/m^2^. In light gray, mean (SE) glucose trajectory for participants with BMI < 25 kg/m^2^. **(B)** Mean glucose trajectory in participants by waist circumference. Results depicted as the mean (SE) glucose concentrations per 5-min interval for a 72-h period. In dark gray, mean (SE) glucose trajectory for participants with the longest waist circumference (>88 cm in women, >102 cm in men). In light gray, mean (SE) glucose trajectory for participants with the shortest waist circumference (≤80 cm in women, ≤94 cm in men).

Graphical representations of the 72 h glucose trajectory in participants with the lowest (<25 kg/m^2^) and highest (>35 kg/m^2^) BMI and shortest (≤80 cm in women; ≤94 cm in men) and longest (>88 cm in women; >102 cm in men) waist circumference are presented in Figure 1.

After meta-analyzing the results from the three cohorts, a higher BMI was associated with a higher 72 h mean glucose concentration, a higher diurnal glucose concentration, and a higher nocturnal glucose level (Table 2). Participants with a BMI between 30 and 35 kg/m^2^ had a 0.28 (95% CI: 0.12, 0.44) mmol/L, higher mean 72 h mean glucose concentration, and a 0.39 (0.19, 0.59) mmol/L higher mean nocturnal glucose concentration as compared with participants with a BMI < 25 kg/m^2^. The observed differences in mean glucose concentrations between the strata were consistently smaller in GOTO compared with AGO and Switchbox, possibly due to the smaller sample size of the GOTO study (Tables S2–S4 in Supplementary Material). Results were similarly observed for men and women (results not shown).

**Table 2:** Associations of measures of adiposity and indices of glycemia in the meta-analyses.

		72-mean glucose (mmol/L)		Diurnal glucose (mmol/L)		Nocturnal glucose (mmol/L)	
	*N*	Mean	Beta (95% CI)	Mean	Beta (95% CI)	Mean	Beta (95% CI)
**Body mass index**							
<25 kg/m^2^	116	5.17	0 (ref)	5.36	0 (ref)	4.40	0 (ref)
25–30 kg/m^2^	216	5.39	0.14 (0.02; 0.27)	5.57	0.12 (−0.01; 0.24)	4.67	0.23 (0.07; 0.38)
30–35 kg/m^2^	86	5.58	0.28 (0.12; 0.44)	5.76	0.26 (0.10; 0.42)	4.87	0.39 (0.19; 0.59)
>35 kg/m^2^	19	6.04	0.85 (0.52; 1.17)	6.27	0.85 (0.52; 1.18)	5.11	0.69 (0.30; 1.07)
**Waist circumference**							
≤80 (W)/≤94 (M) cm	75	5.07	0 (ref)	5.28	0 (ref)	4.31	0 (ref)
80.1–88 (W)/94.1–102 (M) cm	132	5.32	0.22 (0.07; 0.37)	5.51	0.21 (0.06; 0.36)	4.58	0.25 (0.07; 0.44)
>88 (W)/>102 (M) cm	231	5.54	0.42 (0.28; 0.56)	5.73	0.40 (0.26; 0.54)	4.81	0.49 (0.32; 0.67)

*M, men; *N*, number of participants in stratum (all three cohorts combined); W, women*.

*Analyses adjusted for age and sex. Analyses in Switchbox and Growing Old Together additionally corrected for familial relationships. Data presented as difference in outcome (with 95% confidence interval) in millimole per liter with respect to the reference group. “Mean” presents the mean, unadjusted, and glucose concentration in millimole per liter*.

Similarly, a longer waist circumference was also associated with higher levels of glycemia (Table 2). Compared to participants with the shortest waist circumference (≤80 cm in women, ≤94 cm in men), participants with the longest waist circumference (>88 cm in women, >102 cm in men) had a 0.42 mmol/L (0.28, 0.56) higher 72 h mean glucose concentration, a 0.40 mmol/L (0.26, 0.54) higher diurnal glucose concentration, and a 0.49 mmol/L (0.32, 0.67) higher nocturnal glucose concentration. Again, the observed differences in glucose concentrations between the strata were somewhat smaller in GOTO compared with AGO and Switchbox (Tables S2–S4 in Supplementary Material). Results were similarly observed for men and women (results not shown).

### Adiposity and Indices of Glycemic Variability

After meta-analyzing the data of the three cohorts (Table 3), we did not find evidence that a high adiposity level was associated with a higher intraday glycemic variability (as measured with 24-h SD and MAGE), but we found some evidence for a higher between-day glycemic variability (as measured with MODD). Despite the numbers were low (*N* = 19) and all participants originated from AGO, participants with a BMI > 35 kg/m^2^ had a 0.19 units higher MODD (0.02, 0.35) as compared with participants with a BMI < 25 kg/m^2^. Similarly, participants with the longest waist circumference (>88 cm in women, >102 cm in men) had a 0.07 units higher MODD (0.00, 0.14) as compared with participants with the shortest waist circumference (≤80 cm in women, ≤94 cm in men). Results for the intraday variability indices 24 h SD and MAGE were similarly observed in the individual cohorts (Tables S5–S6 in Supplementary Material). However, it should be noted that the associations between waist circumference and MODD were only observed in AGO and GOTO, and not in Switchbox (Table S7 in Supplementary Material). These results were similarly observed when we additionally adjusted for the 72 h mean glucose concentration (results not shown). Results were similarly observed for men and women (results not shown).

**Table 3:** Associations of measures of adiposity and indices of glycemic variability in the meta-analyses.

		SD		mean amplitude of glucose excursion		mean of daily difference	
	*N*	Mean	Beta (95% CI)	Mean	Beta (95% CI)	Mean	Beta (95% CI)
**Body mass index**							
<25 kg/m^2^	116	0.98	0 (ref)	2.36	0 (ref)	0.88	0 (ref)
25–30 kg/m^2^	216	0.93	−0.05 (−0.12; 0.01)	2.20	−0.21 (−0.38; −0.04)	0.85	−0.03 (−0.09; 0.04)
30–35 kg/m^2^	86	0.98	−0.05 (−0.13; 0.03)	2.30	−0.18 (−0.40; 0.03)	0.91	0.03 (−0.05; 0.11)
>35 kg/m^2^	19	1.08	0.09 (−0.09; 0.26)	2.67	0.30 (−0.17; 0.78)	1.06	0.19 (0.02; 0.35)
**Waist circumference**							
≤80 (W)/≤94 (M) cm	75	0.95	0 (ref)	2.30	0 (ref)	0.84	0 (ref)
80.1–88 (W)/94.1–102 (M) cm	132	0.93	−0.01 (−0.09; 0.07)	2.20	−0.06 (−0.27; 0.14)	0.84	0.02 (−0.05; 0.10)
>88 (W)/>102 (M) cm	231	0.98	−0.00 (−0.08; 0.07)	2.33	−0.08 (−0.28; 0.11)	0.91	0.07 (0.00; 0.14)

*M, men; *N*, number of participants in stratum (all three cohorts combined); W, women*.

*Analyses adjusted for age and sex. Analyses in Switchbox and Growing Old Together additionally corrected for familial relationships. Data presented as difference in outcome (with 95% confidence interval) in millimole per liter with respect to the reference group. “Mean” presents the mean, unadjusted, value of the glycemic variability indices*.

## Discussion

Within the present study, we aimed to elaborate on existing knowledge about the association of adiposity and overweight with glycemia in non-diabetic individuals. We observed that, based on data from three independent cohorts, the association between overweight/obesity and glycemia persists over the day, which was reflected by similar associations between overweight/obesity and diurnal, nocturnal and 72-mean glucose concentration. However, we did not find evidence that overweight and/or obesity were associated with a higher intraday glycemic variability, but we found some suggestive evidence, based on a minor subpopulation, that obesity was associated with a higher between-day glycemic variability in individuals with a BMI > 35 kg/m^2^.

Our finding that high adiposity was associated with increased glycemia is in line with the general concept that obesity is a risk factor for developing T2DM (4, 27). Although the precise mechanisms are currently still unclear, adipokines and inflammatory factors might play crucial roles in the pathophysiological mechanisms linking adiposity and T2DM; higher concentrations of IL-6, TNF-α, and leptin expression (8, 9), and lower adiponectin levels (10, 11) associated with increased adiposity levels and insulin resistance, might play a pivotal role in our results as they do in T2DM (28). Future studies, however, are required to study the effect and impact of increased diurnal concentrations of cytokines and adipokines on daily glucose trajectories. Although our findings were, therefore, not unexpected, our findings highlight differences in mean glucose concentrations in the separate adiposity groups remained relatively constant over a 24-h interval (i.e., similar results were observed for diurnal and nocturnal glucose in our study populations). In one of our previous publications on the topic of CGM, we found that genetic variation in the *TCF7L2* gene was predominantly associated with glucose concentration during the nocturnal period (22), which was interpreted as a predominantly hepatic effect. As the present findings were similar for the day and night, the results might be interpreted as a change in set point of the glucose concentration.

The associations between the investigated measures of adiposity and the indices of glycemia were somewhat smaller as compared with the effect sizes observed in AGO and Switchbox, although associations were in similar direction. Potential explanations for this observation could include the different inclusion criteria of the studies. For example, AGO comprised specifically individuals with sedentary behavior and Switchbox and GOTO comprise healthy individuals in which also part of the population was selected for their propensity to become long-lived. Despite having a higher BMI or waist circumference, these observations might suggest that external factors might diminish the higher glucose levels over a 72-h period attributable to adiposity. However, larger (prospective) studies are required to confirm this hypothesis.

In our study population of participants without T2DM, we did not find evidence supporting the existence of an association between higher adiposity and indices of intraday glycemic variability (as measured with 24-h SD and MAGE). These findings are in contrast with a previous study conducted in newly diagnosed T2DM patients where a higher adiposity level was associated with a lower glycemic variability (13). Other studies observed that in patients diagnosed with T2DM, higher glycemic variability has been associated with an increased risk of complications, which includes diabetic retinopathy, cardiovascular autonomic neuropathy, and overall mortality (29, 30). A higher glycemic variability has been shown to be more harmful than high but stable concentrations of glucose; higher glycemic variability results in oxidative stress and endothelial dysfunction, which are two key factors for an increased risk for cardiovascular complications in T2DM patients (31, 32). Although these studies warrant a reduction of glycemic variability, evidence for the importance of glycemic variability in individuals without T2DM is lacking. Based on the results of our study, we hypothesize that the increased glycemia (i.e., increase in set point of glucose concentrations) precedes the increased glycemic variability. However, other explanations, including different biological mechanisms underlying glycemic variability in diabetic and nondiabetic individuals, might explain our results as well. Future prospective studies on the progression of insulin resistance are warranted to confirm this hypothesis.

We additionally found preliminary evidence that participants in the highest BMI/waist circumference category had a higher between-day glycemic variability (as measured with the MODD). Based on this finding, we hypothesize that individuals with high adiposity have a less controlled glycemia, which is reflected by a lower stability of glucose levels between days. However, we acknowledge the small number of individuals in our study with class-II obesity (BMI > 35 kg/m^2^).

The present study has a number of strengths and limitations. The main strength of the present study is that detailed data on daily glucose trajectories (measures every 5 min for a 72-h period) were collected in a large study population (*N* = 438) while the participants were able to pursue their normal daily life activities. Such data provided us with the opportunity to study the effects of adiposity on different indices of glycemia and glycemic variability. These indices have been validated and used before in study populations comprising participants without T2DM (12, 14, 15). However, despite these large numbers, the number of participants with class-II obesity (BMI > 35 kg/m^2^) was still low (*N* = 19). Interpretation of findings based on this group should, therefore, be done with caution, and replication in other (larger) study populations is warranted. The present study was conducted using data collected from three independent study populations with different inclusion criteria and population characteristics. Notably, the AGO study population was recruited with the intention to improve lifestyle (12), and using different in- and exclusion criteria. Participants from Switchbox and GOTO were both enrolled from the Leiden Longevity Study, which included participants based on their propensity to become long-lived together with their partners as controls (17). As the results were relatively similar in the three study populations, this emphasizes the robustness of our findings across populations with different characteristics. Nevertheless, as we conducted an observational study, we need to acknowledge that results could be harmed by residual confounding and/or reverse causation. However, as high adiposity is a known causal risk factor for T2DM and increased levels of glycemia (4), the effect of potential reverse causation was assumed to be absent in our study sample. Furthermore, we were not able to study the effect of the sleep–wake cycle on the results.

In summary, based on the data from our cohorts, we found evidence that increased adiposity is associated with higher levels of glycemia, which remained relatively constant over the day. However, we found no association between increased adiposity and glycemic variability, which questions the importance of glycemic variability in non-diabetic populations. Future studies should elucidate on the potential biological contributors to our observed phenotype.

## Clinical Trial Registration

The AGO study was registered in the Dutch Trial Register (http://www.trialregister.nl) as NTR3045. The GOTO study was registered in the Dutch Trial Register (http://www.trialregister.nl) as NTR3499.

## Ethics Statement

The three studies were carried out in accordance with the recommendations of the Helsinki Declaration. The protocol was approved by the medical ethical committee of the Leiden University Medical Center (LUMC), Leiden, the Netherlands. All three studies were performed within the LUMC. Written informed consent was obtained from all study participants.

## Author Contributions

Study design: RN, NH, PS, SM, and DH. Data collection: NH, CW, AA, SJ, SS, MB, OR, PS, SM, and DH. Data analyses: RN and NH. Interpretation of the data: RN, NH, and DH. Drafting the manuscript: RN and NH. Editing of the initial versions of the manuscript: NH, CW, AA, SH, SS, MB, OR, PS, SM, and DH. Final approval: RN, NH, CW, AA, SJ, SS, MB, OR, PS, SM, and DvH.

## Conflict of Interest Statement

The authors declare that the research was conducted in the absence of any commercial or financial relationships that could be construed as a potential conflict of interest.
