# Supplementary material for: High Adiposity Is Associated With Higher Nocturnal and Diurnal Glycaemia, but Not With Glycemic Variability in Older Individuals Without Diabetes
Source: Front Endocrinol (Lausanne). 2018 May 14;9:238. doi: 10.3389/fendo.2018.00238 (PMC5960684; doi:10.3389/fendo.2018.00238)
Supplement: Supplementary file 1 [file Table_1.docx]

| **Supplementary Table 1:** Characteristics of the three study populations | | | |
| --- | --- | --- | --- |
|  | AGO (N=228) | Switchbox (N=116) | GOTO (N=94) |
| **Demographics** |  |  |  |
| Age (years), mean (SD) | 64.8 (2.9) | 65.7 (6.2) | 63.4 (5.4) |
| Men, n (%) | 137 (60.1) | 49 (52.1) | 56 (48.3) |
|  |  |  |  |
| **Body composition** |  |  |  |
| Body mass index (kg/m^2^), mean (SD) | 28.9 (4.5) | 26.0 (4.1) | 26.8 (2.3) |
| Waist circumference (cm), mean (SD)^1^ | 101.7 (12.3) | 93.9 (12.6) | 95.8 (8.0) |
|  |  |  |  |
| **Measurements derived with CGM** |  |  |  |
| 72-h mean glucose (mmol/L), mean (SD) | 5.5 (0.6) | 5.3 (0.5) | 5.2 (0.5) |
| Nocturnal glucose (mmol/L), mean (SD) | 4.8 (0.7) | 4.6 (0.7) | 4.4 (0.6) |
| Diurnal glucose (mmol/L), mean (SD) | 5.7 (0.7) | 5.5 (0.5) | 5.4 (0.5) |
| MAGE, mean (SD) | 2.3 (0.9) | 2.3 (0.6) | 2.2 (0.7) |
| 72-h SD, mean (SD) | 1.0 (0.3) | 0.9 (0.2) | 0.9 (0.3) |
| MODD, mean (SD) | 0.9 (0.3) | 0.9 (0.3) | 0.8 (0.2) |

Abbreviations: AGO, “Actief en Gezond Oud”; GOTO, Growing Old Together; MAGE, mean amplitude of glycaemic excursions; MODD, mean of daily differences; SD, standard deviation. ^1^ Missing for two participants in the AGO Study.
