# Supplementary material for: High Adiposity Is Associated With Higher Nocturnal and Diurnal Glycaemia, but Not With Glycemic Variability in Older Individuals Without Diabetes
Source: Front Endocrinol (Lausanne). 2018 May 14;9:238. doi: 10.3389/fendo.2018.00238 (PMC5960684; doi:10.3389/fendo.2018.00238)
Supplement: Supplementary file 2 [file Table_2.docx]

| **Supplementary Table 2:** Associations of measures of adiposity and 72-mean glucose concentration in the individual cohorts | | | | | | | | | | | | |
| --- | --- | --- | --- | --- | --- | --- | --- | --- | --- | --- | --- | --- |
|  |  | AGO | | |  | Switchbox | | |  | GOTO | | |
|  |  | N | Mean | Beta (95% CI) |  | N | Mean | Beta (95% CI) |  | N | Mean | Beta (95% CI) |
| **Body mass index** |  |  |  |  |  |  |  |  |  |  |  |  |
| < 25 kg/m^2^ |  | 37 | 5.18 | 0 (ref) |  | 53 | 5.16 | 0 (ref) |  | 26 | 5.16 | 0 (ref) |
| 25-30 kg/m^2^ |  | 114 | 5.47 | 0.18 (-0.04; 0.40) |  | 42 | 5.37 | 0.18 (-0.03; 0.38) |  | 60 | 5.24 | 0.06 (-0.17; 0.29) |
| 30-35 kg/m^2^ |  | 58 | 5.68 | 0.38 (0.14; 0.63) |  | 20 | 5.43 | 0.26 (0.01; 0.51) |  | 8 | 5.19 | 0.07 (-0.32; 0.46) |
| >35 kg/m^2^ |  | 19 | 6.04 | 0.85 (0.52; 1.17) |  | 1 | - | NA |  | 0 | - | NA |
|  |  |  |  |  |  |  |  |  |  |  |  |  |
| **Waist circumference** |  |  |  |  |  |  |  |  |  |  |  |  |
| ≤80 (W) / ≤94 (M) cm |  | 23 | 5.11 | 0 (ref) |  | 36 | 5.05 | 0 (ref) |  | 16 | 5.08 | 0 (ref) |
| 80.1–88 (W) / 94.1–102 (M) cm |  | 63 | 5.37 | 0.19 (-0.09; 0.47) |  | 32 | 5.34 | 0.29 (0.06; 0.51) |  | 37 | 5.21 | 0.15 (-0.14; 0.43) |
| >88 (W) / >102 (M) cm |  | 142 | 5.66 | 0.55 (0.29; 0.80) |  | 48 | 5.43 | 0.39 (0.18; 0.59) |  | 41 | 5.27 | 0.31 (0.01; 0.61) |

Abbreviations: M, men; N, number of participants in stratum (all three cohorts combined); W, women. Analyses adjusted for age and sex. Analyses in Switchbox and GOTO additionally corrected for familial relationships. Data presented as difference in outcome (with 95% confidence interval) in mmol/L with respect to the reference group. “Mean” presents the mean glucose concentration in mmol/L.
