# Supplementary material for: High Adiposity Is Associated With Higher Nocturnal and Diurnal Glycaemia, but Not With Glycemic Variability in Older Individuals Without Diabetes
Source: Front Endocrinol (Lausanne). 2018 May 14;9:238. doi: 10.3389/fendo.2018.00238 (PMC5960684; doi:10.3389/fendo.2018.00238)
Supplement: Supplementary file 3 [file Table_3.docx]

| **Supplementary Table 3:** Associations of measures of adiposity and diurnal glucose concentration in the individual cohorts | | | | | | | | | | | | |
| --- | --- | --- | --- | --- | --- | --- | --- | --- | --- | --- | --- | --- |
|  |  | AGO | | |  | Switchbox | | |  | GOTO | | |
|  |  | N | Mean | Beta (95% CI) |  | N | Mean | Beta (95% CI) |  | N | Mean | Beta (95% CI) |
| **Body mass index** |  |  |  |  |  |  |  |  |  |  |  |  |
| < 25 kg/m^2^ |  | 37 | 5.36 | 0 (ref) |  | 53 | 5.35 | 0 (ref) |  | 26 | 5.38 | 0 (ref) |
| 25-30 kg/m^2^ |  | 114 | 5.65 | 0.15 (-0.08; 0.38) |  | 42 | 5.54 | 0.15 (-0.06; 0.35) |  | 60 | 5.44 | 0.03 (-0.21; 0.28) |
| 30-35 kg/m^2^ |  | 58 | 5.87 | 0.36 (0.10; 0.61) |  | 20 | 5.63 | 0.26 (0.00; 0.51) |  | 8 | 5.35 | 0.02 (-0.39; 0.43) |
| >35 kg/m^2^ |  | 19 | 6.27 | 0.85 (0.52; 1.18) |  | 1 | - | NA |  | 0 | - | NA |
|  |  |  |  |  |  |  |  |  |  |  |  |  |
| **Waist circumference** |  |  |  |  |  |  |  |  |  |  |  |  |
| ≤80 (W) / ≤94 (M) cm |  | 23 | 5.29 | 0 (ref) |  | 36 | 5.26 | 0 (ref) |  | 16 | 5.30 | 0 (ref) |
| 80.1–88 (W) / 94.1–102 (M) cm |  | 63 | 5.55 | 0.18 (-0.11; 0.47) |  | 32 | 5.53 | 0.28 (0.05; 0.50) |  | 37 | 5.42 | 0.14 (-0.16; 0.44) |
| >88 (W) / >102 (M) cm |  | 142 | 5.85 | 0.56 (0.30; 0.83) |  | 48 | 5.60 | 0.35 (0.15; 0.56) |  | 41 | 5.46 | 0.28 (-0.04; 0.59) |

Abbreviations: M, men; N, number of participants in stratum (all three cohorts combined); W, women. Analyses adjusted for age and sex. Analyses in Switchbox and GOTO additionally corrected for familial relationships. Data presented as difference in outcome (with 95% confidence interval) in mmol/L with respect to the reference group. “Mean” presents the mean glucose concentration in mmol/L.
