# Supplementary material for: High Adiposity Is Associated With Higher Nocturnal and Diurnal Glycaemia, but Not With Glycemic Variability in Older Individuals Without Diabetes
Source: Front Endocrinol (Lausanne). 2018 May 14;9:238. doi: 10.3389/fendo.2018.00238 (PMC5960684; doi:10.3389/fendo.2018.00238)
Supplement: Supplementary file 4 [file Table_4.docx]

| **Supplementary Table 4:** Associations of measures of adiposity and nocturnal glucose concentration in the individual cohorts | | | | | | | | | | | | |
| --- | --- | --- | --- | --- | --- | --- | --- | --- | --- | --- | --- | --- |
|  |  | AGO | | |  | Switchbox | | |  | GOTO | | |
|  |  | N | Mean | Beta (95% CI) |  | N | Mean | Beta (95% CI) |  | N | Mean | Beta (95% CI) |
| **Body mass index** |  |  |  |  |  |  |  |  |  |  |  |  |
| < 25 kg/m^2^ |  | 37 | 4.42 | 0 (ref) |  | 53 | 4.41 | 0 (ref) |  | 26 | 4.34 | 0 (ref) |
| 25-30 kg/m^2^ |  | 114 | 4.75 | 0.31 (0.05; 0.58) |  | 42 | 4.75 | 0.33 (0.04; 0.61) |  | 60 | 4.45 | 0.07 (-0.19; 0.32) |
| 30-35 kg/m^2^ |  | 58 | 4.97 | 0.52 (0.23; 0.82) |  | 20 | 4.70 | 0.28 (-0.06; 0.63) |  | 8 | 4.54 | 0.25 (-0.20; 0.69) |
| >35 kg/m^2^ |  | 18 | 5.11 | 0.69 (0.30; 1.07) |  | 1 | - | NA |  | 0 | - | NA |
|  |  |  |  |  |  |  |  |  |  |  |  |  |
| **Waist circumference** |  |  |  |  |  |  |  |  |  |  |  |  |
| ≤80 (W) / ≤94 (M) cm |  | 23 | 4.40 | 0 (ref) |  | 36 | 4.28 | 0 (ref) |  | 16 | 4.25 | 0 (ref) |
| 80.1–88 (W) / 94.1–102 (M) cm |  | 63 | 4.67 | 0.25 (-0.09; 0.59) |  | 32 | 4.60 | 0.32 (0.01; 0.63) |  | 37 | 4.42 | 0.19 (-0.13; 0.50) |
| >88 (W) / >102 (M) cm |  | 142 | 4.90 | 0.50 (0.19; 0.81) |  | 48 | 4.80 | 0.53 (0.25; 0.81) |  | 41 | 4.50 | 0.43 (0.10; 0.77) |

Abbreviations: M, men; N, number of participants in stratum (all three cohorts combined); W, women. Analyses adjusted for age and sex. Analyses in Switchbox and GOTO additionally corrected for familial relationships. Data presented as difference in outcome (with 95% confidence interval) in mmol/L with respect to the reference group. “Mean” presents the mean glucose concentration in mmol/L.
