# Supplementary material for: High Adiposity Is Associated With Higher Nocturnal and Diurnal Glycaemia, but Not With Glycemic Variability in Older Individuals Without Diabetes
Source: Front Endocrinol (Lausanne). 2018 May 14;9:238. doi: 10.3389/fendo.2018.00238 (PMC5960684; doi:10.3389/fendo.2018.00238)
Supplement: Supplementary file 5 [file Table_5.docx]

| **Supplementary Table 5:** Associations of measures of adiposity and 72-h standard deviation in the individual cohorts | | | | | | | | | | | | |
| --- | --- | --- | --- | --- | --- | --- | --- | --- | --- | --- | --- | --- |
|  |  | AGO | | |  | Switchbox | | |  | GOTO | | |
|  |  | N | Mean | Beta (95% CI) |  | N | Mean | Beta (95% CI) |  | N | Mean | Beta (95% CI) |
| **Body mass index** |  |  |  |  |  |  |  |  |  |  |  |  |
| < 25 kg/m^2^ |  | 37 | 0.98 | 0 (ref) |  | 53 | 0.97 | 0 (ref) |  | 26 | 1.01 | 0 (ref) |
| 25-30 kg/m^2^ |  | 114 | 0.94 | -0.07 (-0.19; 0.05) |  | 42 | 0.91 | -0.07 (-0.17; 0.04) |  | 60 | 0.91 | -0.10 (-0.22; 0.02) |
| 30-35 kg/m^2^ |  | 58 | 1.01 | 0.00 (-0.13; 0.14) |  | 20 | 0.94 | -0.04 (-0.16; 0.08) |  | 8 | 0.81 | -0.19 (-0.40; 0.01) |
| >35 kg/m^2^ |  | 19 | 1.08 | 0.09 (-0.09; 0.26) |  | 1 | - | NA |  | 0 | - | NA |
|  |  |  |  |  |  |  |  |  |  |  |  |  |
| **Waist circumference** |  |  |  |  |  |  |  |  |  |  |  |  |
| ≤80 (W) / ≤94 (M) cm |  | 23 | 0.91 | 0 (ref) |  | 36 | 0.98 | 0 (ref) |  | 16 | 0.94 | 0 (ref) |
| 80.1–88 (W) / 94.1–102 (M) cm |  | 63 | 0.92 | -0.01 (-0.16; 0.15) |  | 32 | 0.97 | -0.01 (-0.12; 0.11) |  | 37 | 0.91 | -0.02 (-0.17; 0.14) |
| >88 (W) / >102 (M) cm |  | 142 | 1.01 | 0.10 (-0.04; 0.24) |  | 48 | 0.90 | -0.07 (-0.17; 0.04) |  | 41 | 0.95 | 0.02 (-0.14; 0.18) |

Abbreviations: M, men; N, number of participants in stratum (all three cohorts combined); W, women. Analyses adjusted for age and sex. Analyses in Switchbox and GOTO additionally corrected for familial relationships. Data presented as difference in outcome (with 95% confidence interval) with respect to the reference group. “Mean” presents the mean SD.
