# Supplementary material for: High Adiposity Is Associated With Higher Nocturnal and Diurnal Glycaemia, but Not With Glycemic Variability in Older Individuals Without Diabetes
Source: Front Endocrinol (Lausanne). 2018 May 14;9:238. doi: 10.3389/fendo.2018.00238 (PMC5960684; doi:10.3389/fendo.2018.00238)
Supplement: Supplementary file 6 [file Table_6.docx]

| **Supplementary Table 6:** Associations of measures of adiposity and the mean amplitude of glycaemic excursions in the individual cohorts | | | | | | | | | | | | |
| --- | --- | --- | --- | --- | --- | --- | --- | --- | --- | --- | --- | --- |
|  |  | AGO | | |  | Switchbox | | |  | GOTO | | |
|  |  | N | Mean | Beta (95% CI) |  | N | Mean | Beta (95% CI) |  | N | Mean | Beta (95% CI) |
| **Body mass index** |  |  |  |  |  |  |  |  |  |  |  |  |
| < 25 kg/m^2^ |  | 37 | 2.34 | 0 (ref) |  | 53 | 2.36 | 0 (ref) |  | 26 | 2.41 | 0 (ref) |
| 25-30 kg/m^2^ |  | 114 | 2.23 | -0.19 (-0.52; 0.14) |  | 42 | 2.18 | -0.20 (-0.46; 0.06) |  | 60 | 2.17 | -0.24 (-0.56; 0.08) |
| 30-35 kg/m^2^ |  | 58 | 2.40 | -0.02 (-0.38; 0.34) |  | 20 | 2.19 | -0.19 (-0.51; 0.13) |  | 8 | 1.84 | -0.54 (-1.09; 0.01) |
| >35 kg/m^2^ |  | 19 | 2.67 | 0.30 (-0.17; 0.78) |  | 1 | - | NA |  | 0 | - | NA |
|  |  |  |  |  |  |  |  |  |  |  |  |  |
| **Waist circumference** |  |  |  |  |  |  |  |  |  |  |  |  |
| ≤80 (W) / ≤94 (M) cm |  | 23 | 2.25 | 0 (ref) |  | 36 | 2.38 | 0 (ref) |  | 16 | 2.21 | 0 (ref) |
| 80.1–88 (W) / 94.1–102 (M) cm |  | 63 | 2.16 | -0.14 (-0.55; 0.27) |  | 32 | 2.35 | -0.02 (-0.32; 0.27) |  | 37 | 2.13 | -0.06 (-0.47; 0.36) |
| >88 (W) / >102 (M) cm |  | 142 | 2.42 | 0.17 (-0.21; 0.55) |  | 48 | 2.11 | -0.26 (-0.53; 0.01) |  | 41 | 2.27 | 0.06 (-0.38; 0.50) |

Abbreviations: M, men; N, number of participants in stratum (all three cohorts combined); W, women. Analyses adjusted for age and sex. Analyses in Switchbox and GOTO additionally corrected for familial relationships. Data presented as difference in outcome (with 95% confidence interval) with respect to the reference group. “Mean” presents the mean “mean amplitude of glycaemic excursions”.
